# The Role of a Social Worker in the Deep Brain Stimulation Preoperative Evaluation: The DBS‐FACTS Screening Tool

**DOI:** 10.1002/mdc3.70218

**Published:** 2025-07-11

**Authors:** Anne E.P. Mulders, Colin van der Heijden, Mark L. Kuijf, Rianne Esselink, Annelien A. Duits

**Affiliations:** ^1^ Department of Medical Psychology Maastricht University Medical Center+ Maastricht The Netherlands; ^2^ Department of Medical Psychology Radboud University Medical Center Nijmegen The Netherlands; ^3^ Department of Neurology Maastricht University Medical Center+ Maastricht The Netherlands; ^4^ Department of Neurology Radboud University Medical Center, Donders Institute for Brain, Cognition and Behaviour Nijmegen The Netherlands

With great interest we read the recent article by Currie et al.^1^ that describes the role of social workers in the preoperative evaluation for deep brain stimulation (DBS) and introduces the DBS‐FACTS screening tool to identify patients who may benefit from comprehensive social work consultation during the DBS screening and evaluation process. We are enthusiastic about the development of this tool and believe it has the potential to optimize the use of social work resources and thereby improve overall well‐being of patients who receive DBS.

Based on our clinical experience and an exploratory project aimed at developing a psychosocial care program for Parkinson's disease (PD) patients undergoing DBS surgery,^2^ we would like to propose an addition to the suggested screening tool: expectation management.

As part of our project called “Beter in Balans” (“better balanced”),^2^ we explored the experiences and needs related to DBS in PD from multiple perspectives. We conducted 2 focus group interviews with PD patients who had undergone DBS—along with their spouses or informal caregivers when available—and we interviewed DBS professionals from all DBS centers in the Netherlands, including nurses, neurosurgeons, social workers, neurologists, and psychologists. Across all groups, both professionals and patients consistently emphasized the importance of managing expectations in the preoperative phase.

Unrealistic expectations regarding the outcomes of DBS can negatively impact a patient's satisfaction and subjective well‐being after surgery^3^ and that of his or her informal caregivers. Importantly, expectations often extend beyond symptom reduction and may include broader goals such as improved participation in work and family life and engagement in hobbies. Moreover, patients may underestimate potential side effects, the time needed to optimize stimulation settings postoperatively, and the continued progression of PD. When unrealistic expectations are identified, referral to a social worker should be considered, as they can play a key role in addressing and managing these diverse themes, preferably prior to surgery. Moreover, the social worker advises, informs, and involves other disciplines form the DBS team if this contributes to managing expectations.

At our DBS centers (Radboud and Maastricht University Medical Center), we have adopted a similar approach to that proposed by Currie et al.^1^ However, we also explicitly assess the patient's current coping strategies and expectations during the preoperative phase and involve the social worker if believed necessary. Postoperatively, the social worker may also support the patient and his or her family network in dealing with changes related to the emotional impact of the surgery, and shifts in physical functioning, roles, and identity, as well as in recognizing and addressing potential nonmotor side effects or coping challenges.

We would suggest using the term “DBS‐EXFACTS” to emphasize the critical role of managing patient expectations alongside the existing domains. We believe that this integrated approach, in which the social worker plays a central role in managing expectations and supporting emotional and psychosocial adjustment, helps to mitigate disappointment and contributes significantly to patient well‐being throughout the DBS journey.

## Author Roles

1. Research project: A. Conception, B. Organization, C. Execution;
2. Statistical analysis: A. Design, B. Execution, C. Review and critique;
3. Manuscript preparation: A. Writing of the first draft, B. Review and critique.

A.E.P.M.: 3A

C.V.H.: 3B

M.L.K.: 3B

R.E.: 3B

A.A.D.: 3B

## Disclosures

**Ethical Compliance Statement**: Ethical approval for this study was provided by the Maastricht University Medical Center Ethical Committee because of non‐WMO research 2019‐1032. Written informed consent was obtained from all focus group participants. We confirm that we have read the journal's position on issues involved in ethical publication and affirm that this work is consistent with those guidelines.

**Funding Sources and Conflicts of Interest**: The “Beter in Balans” project was funded by Parkinson NL (P2019‐12).

The authors declare that there are no conflicts of interest relevant to this work.

**Financial Disclosures for the Previous 12 Months**: A.E.P.M, C.V.H, M.L.K. and R.E. have no additional disclosures to report. A.A.D. received research grants from the following organizations: Radboudfonds and the Health Foundation Limburg.

## Data Availability

The data that support the findings of this study are available from the corresponding author upon reasonable request.
